# A thrombophilic allele of clotting Factor VII/VIIa promoting recurrent pulmonary emboli, clinical details, and a structural model of the altered protein: a case report

**DOI:** 10.1186/s13256-023-03833-0

**Published:** 2023-04-13

**Authors:** Kenneth Newman, Fevzi Daldal, Andrew Dancis

**Affiliations:** 1 Department of Medicine, Corporal Michael J. Crescenz VAMC, 3900 Woodland Avenue, Philadelphia, PA 19104 USA; 2 Department of Biology, University of Pennsylvania, Philadelphia, PA 19104 USA

**Keywords:** Factor VII, Factor VIIa, Tissue Factor (TF), Zymogen, Thrombophilia, Hemostasis, DOAC

## Abstract

**Background:**

The clotting or hemostasis system is a meticulously regulated set of enzymatic reactions that occur in the blood and culminate in formation of a fibrin clot. The precisely calibrated signaling system that prevents or initiates clotting originates with the activated Factor Seven (FVIIa) complexed with tissue factor (TF) formed in the endothelium. Here we describe a rare inherited mutation in the FVII gene which is associated with pathological clotting.

**Case presentation:**

The 52-year-old patient, with European, Cherokee and African American origins, FS was identified as having low FVII (10%) prior to elective surgery for an umbilical hernia. He was given low doses of NovoSeven (therapeutic Factor VIIa) and had no unusual bleeding or clotting during the surgery. In fact, during his entire clinical course he had no unprovoked bleeding. Bleeding instances occurred with hemostatic stresses such as gastritis, kidney calculus, orthopedic surgery, or tooth extraction, and these were handled without factor replacement. On the other hand, FS sustained two unprovoked and life-threatening instances of pulmonary emboli, although he was not treated with NovoSeven at any time close to the events. Since 2020, he has been placed on a DOAC (Direct Oral Anticoagulant, producing Factor Xa inhibition) and has sustained no further clots.

**Possible mechanism of (unauthorized) FVII activation:**

FS has a congenitally mutated FVII/FVIIa gene, which carries a R315W missense mutation in one allele and a mutated start codon (ATG to ACG) in the other allele, thus rendering the patient effectively homozygous for the missense FVII. Structure based comparisons with known crystal structures of TF-VIIa indicate that the patient's missense mutation is predicted to induce a conformational shift of the C170's loop due to crowding of the bulky tryptophan to a distorted "out" position (Fig. 1). This mobile loop likely forms new interactions with activation loop 3, stabilizing a more active conformation of the FVII and FVIIa protein. The mutant form of FVIIa may be better able to interact with TF, displaying a modified serine protease active site with enhanced activity for downstream substrates such as Factor X.

**Conclusions:**

Factor VII can be considered the gatekeeper of the coagulation system. Here we describe an inherited mutation in which the gatekeeper function is altered. Instead of the expected bleeding manifestations resulting from a clotting factor deficiency, the patient FS suffered clotting episodes. The efficacy of the DOAC in treating and preventing clots in this unusual situation is due to its target site of inhibition (anti-Xa), which lies downstream of the site of action of FVIIa/TF.

## Background

Activated Factor VIIa is the initiator of the so-called extrinsic clotting cascade. However, its activity is generally inhibited by a double check, being maintained in a zymogen form and being held in an inactive conformation Bernardi and Mariani [1]. Only when the integrity of the circulatory system is disrupted and interaction with tissue factor (TF) cofactor occurs is the FVII procoagulant potential unveiled. Tissue factor is usually sequestered outside the circulatory system in the subendothelial space, and TF only "sees" FVII/VIIa if the endothelial barrier is violated. FVIIa is a serine protease clotting factor present in the circulation primarily as an inactive zymogen. The FVII zymogen circulates at low concentrations (500 ng/ml or 5 nM in human plasma). The extracellular cleavage of a small portion, of FVII gives rise to VIIa (about 1% or 0.1 nM in human plasma), occurring between residues Arg152 and Ile 153 and producing FVII heavy and light chains. The serine protease activity of the enzyme in the heavy chain is still masked, however, until interaction with TF occurs [1]. This protein–protein interaction occurs over a large surface area and resembles an embrace, with contact sites in all of the various domains of the elongated FVII/VIIa structure (Banner *et al.*, Nature, 380:41-46, 1996). In the presence of an appropriate lipid environment such as activated phospholipid membranes, the allosteric changes in FVII/VIIa increase the protease activity up to 10^sixfold for the physiologic substrates Factor X and Factor IX as well as autocatalytic activation of FVII [2]. Once activated, FVIIa turns on a cascade of proteases termed the extrinsic clotting system, including FX, prothrombin and thrombin, subsequently cleaving fibrinogen to fibrin and making a clot polymer [2].

The F7 gene (12.8 kb) is located on chromosome 13q32 and gives rise to three messenger RNA (mRNA) transcripts through alternative splicing. The two transcripts NM_019616.4 and NM_000131.4 encode an identical mature FVII protein. The transcripts are translated in cells of various tissues especially in liver, and the 466 amino acid protein includes 60 amino terminal amino acids that constitute the prepropeptide. This is removed by intracellular proteolysis during processing and secretion steps. Thus, the mature circulating FVII (50 kDa) consists of 406 residues lacking the precursor segment. The mature zymogen including amino acids 1–406 circulates at low concentrations in the blood, and a small amount is processed by cleavage of FVIIa into heavy and light chains. The heavy chain contains the serine protease domain, and the light chain contains the calcium binding, vitamin K dependent (GLA) and two epidermal growth factor like domains needed for interactions with membranes and other proteins of the clotting cascade. The cleaved form of FVIIa, though, including heavy and light chains, is still largely inactive until exposure to TF occurs [2].

Congenital FVII deficiency (OMIM 227500) has been defined as a bleeding disorder associated with FVII coagulant activity below 50% of normal. However, bleeding is rarely seen unless the FVII coagulant activity falls below 10%. Even in such cases, there is a poor correlation of the factor level with bleeding predisposition [3]. The bleeding phenotype may include central nervous system bleeding in the most severe forms, gastrointestinal bleeding, hemarthrosis, or in mild forms only mucocutaneous bleeding and bruising. Instances of operative bleeding or post-op bleeding have been described associated with low FVII coagulant activity. The reason for the poor correlation of FVII activity levels with bleeding is unclear. Levels above 26% are rarely associated with problems, and so this may be considered a safe threshold for therapy. In contrast, individuals with levels less than 10% may bleed spontaneously. Note that an extensive database of FVII mutations exists [4], and these include promoter mutations, splice site mutations, missense mutations in the various critical protein domains. Null alleles are absent from the database, because the F7 gene is essential for organism viability [1]. A specific mutant allele has not been shown to predict for bleeding or other phenotypes. Recombinant FVIIa (NovoSeven) is a pharmaceutical that has been effectively utilized to prevent or arrest bleeding in the setting of severe FVII deficiency [3]. Its optimal efficacy has been achieved with very low doses such as 15 µg/kg, rather than with the higher doses used for bypassing FVIII inhibitors.

A number of individuals with FVII deficiency have been described that experienced clotting manifestations rather than bleeding manifestations, such as venous thromboembolism or pulmonary embolism. These events occurred in the setting of deficient FVII activity, leading to the conclusion that deficiency of this essential clotting factor does not protect against pathologic clotting [5]. A related and unanswered question is whether some FVII mutant alleles may predispose to clots. In a paper by Mariani *et al.* [5], nine individuals were described with FVII deficiency and clots. The clots were arterial in one case (a vascular lesion in the striatum) and venous in seven (deep vein thrombosis, superficial venous thrombosis and pulmonary embolism). The remaining patient had overt disseminated intravascular coagulation. Two patients experienced spontaneous thrombotic episodes (pulmonary embolism and an arterial cerebral lesion) while in six cases, the thrombotic events where apparently secondary to surgical interventions or delivery (one case). The specific alleles identified included the A294V missense in 6 cases, some of which were heterozygous, some of which were homozygous. This A294V mutant residue might be shifted from the zymogen to the FVIIa conformation by the mutation, although this has not been established. The C310P mutation occurred as a heterozygous change in an individual with no bleeding but with a deep vein thrombosis. This mutation is expected to disrupt the 310–329 disulfide bridge in the C170's loop [5]. Its location in the protein is nearby to the R315W mutation in our patient, also located in the C170's loop.

The R315W FVII gene substitution appearing in our patient FS has been previously reported and characterized [6]. The published case was quite different from ours. The patient in the prior publication was doubly heterozygous for the F315W mutation and for an R304Q substitution. This patient had slightly decreased FVII activity (52%) and more severely decreased activity towards FX in plasma (34%), however he did not experience instances of bleeding after hemostatic stress. He never experienced any clots or thrombosis. In the published paper, the authors described the in vitro expression of the R315W mutant form of FVII. They found that it exhibited enzyme activity albeit somewhat decreased, and furthermore the mutant form showed a shorter half-life and decreased recovery, consistent with alterations in protein folding.

**Fig. 1:**
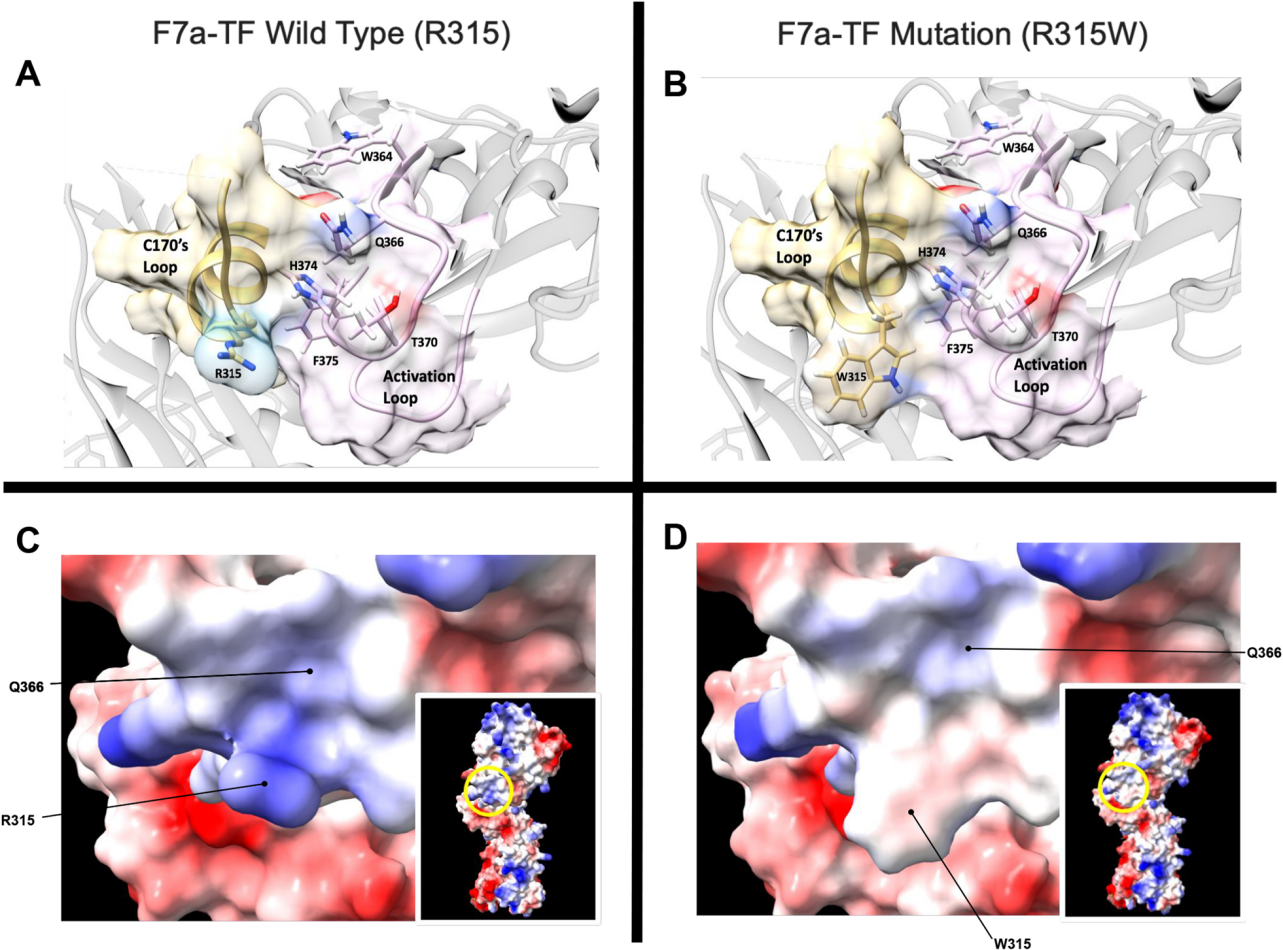
Structural changes predicted to be induced by the R315W mutation. The FVIIa-TF complex (PDB 1DAN) is depicted using UCSF Chimera X [14] as a ribbon structure (panel **A**, light gray) where the critical residues are labeled (numbering refers to the mature processed form) and shown as ball and stick representations with blue and red for basic and acidic chemical groups, respectively. The structure is oriented such that the R315 region faces out, and the critical features are overlayed with a space-filling representation, where the C170's loop (aa 310–329) shaded in light yellow is adjacent and partially overlapping with the alpha 2 helix (aa 306–313), the protruding R315 region is in light blue, and the activation loop 3 (aa 365–374) [11] is in pink. A similar structural model corresponding to the mutated FVIIa-TF is computationally constructed using the DUET [15], a predictive server that models missense mutations. We replaced the R315 with W to depict the mutation found in our patient (FS) (panel **B**). The two models are shown in the same orientation (with respect to R315) for easy comparison, and the specific residues and features are maintained as in panel **A**, except for the W315 and its surrounding region. We utilized the Dunbrack 2010 backbone-dependent rotamer library [16] to estimate the preferred rotamer conformation for our residue of interest. The missense tryptophan 315 was found to prefer an "outward" orientation. The substitution of the R315 with its basic chemical group by a neutral and bulkier W drastically affected the local conformation and electrostatics around the C170's loop due to loss of charge and increased side chain volume. In the bottom part of the figure (panels **C** and **D**), the local changes are better visualized using a space-filling model depicting the local surface charges (blue for positive and red for negative) of wild type and mutant FVIIa R315 (panel **C**) and mutant W315 (panel **D**). The insets depict the entire VIIa-TF complex for context, with the encircled regions highlighting the regions of interest for the wild type R315 and mutant W315 areas

The key to the function of FVII/VIIa is that it is activated by interaction with its cofactor TF. Major structural and allosteric changes occur as a consequence of this interaction leading to modification of the shape of the serine protease site and increases in affinity and activity for downstream substrates [2]. The structure of the binary complex (TF-VIIa) was determined in 1996 [7] but the conformational effects of cofactor binding to VIIa could not be ascertained because of lack of structural information on free factor VIIa. This deficit was remedied in 1999 by the publication of the structure of the gamma carboxyglutamic acid-domainless human VIIa at 2.8 Angstroms [8]. The VIIa molecule adopts an extended conformation within the crystal with separate GLA, EGF1, EGF2 and protease domains, easily distinguished in the structure. The binding mode of the active site inhibitor D-Phe-Phe-Arg methyl ketone differs in the two structures, suggesting a role for the TF cofactor in altering the substrate recognition site. Importantly a surface exposed alpha helix in the C170's loop in the protease domain (amino acids 307–312) which is located at the cofactor recognition site is distorted in the free form [8]. The precise molecular switch by which TF turns on FVIIa remains elusive but a good guess is that this alpha helix functions as the critical switch, conveying a signal to the activation loop 3 and thence to the substrate binding site. Note that the FVII R315W amino acid resides in the same C170's loop adjacent to this alpha helix. Our hypothesis is that the amino acid substitution modifies the loop configuration and may mimic the effect of TF binding or may enhance TF binding, thereby activating the entire intrinsic clotting pathway (Fig. 1).

## Case presentation

Diagnosis and first surgery with prophylactic treatment. In October 2013, Mr. FS was a 52 year old man, with European, Cherokee and African American origins, who presented to our Hematology clinic, prior to a planned umbilical hernia repair. The hernia was a rather large umbilical hernia, but it was not incarcerated, and the planned surgery was more cosmetic than emergent. Routine pre-op workup at the Hospital of the University of Pennsylvania William Pepper Laboratory revealed a prolonged PT of 20.6 s and a normal PTT of 27.7 s. The PT corrected with 1:1 mixing with normal plasma, and incubation at 37 degrees after mixing for 30 min did not result in PT prolongation, showing that an inhibitor was not present. The precise factor deficiency was ascertained by mixes with various factor deficient plasmas: mixing with Factor II, V, or X deficient plasma did correct the PT prolongation, whereas mixing with Factor VII deficient plasma did not correct. The conclusion was that the patient was deficient in Factor VII with a measured level of FVII activity of 10% (66–150% normal range). With this information, we returned to the patient and inquired about his prior bleeding and clotting history. In fact, he had no history of bruising or unusual bleeding events even in the setting of hemostatic stresses. He had never been transfused with blood products. He also never had any abnormal clotting (these events occurred later). More specifically: he had experienced several hemostatic stresses including repair of hemorrhoids 9 years ago, repair of an anal fissure 6 years ago, tooth extractions 6 years ago, treatment of a bleeding ulcer that required cauterization 6 years go. The level of FVII activity correlates poorly with bleeding tendency [9], and since the level was quite low (5–10%), we elected to treat prophylactically with NovoSeven (therapeutic activated Factor VIIa), albeit using an extremely low dose of 1 mg prior to the surgery and 1 mg 4 h post op [3]. No problems were encountered during the surgery, and no unusual bleeding or clotting ensued.

**Fig. 2:**
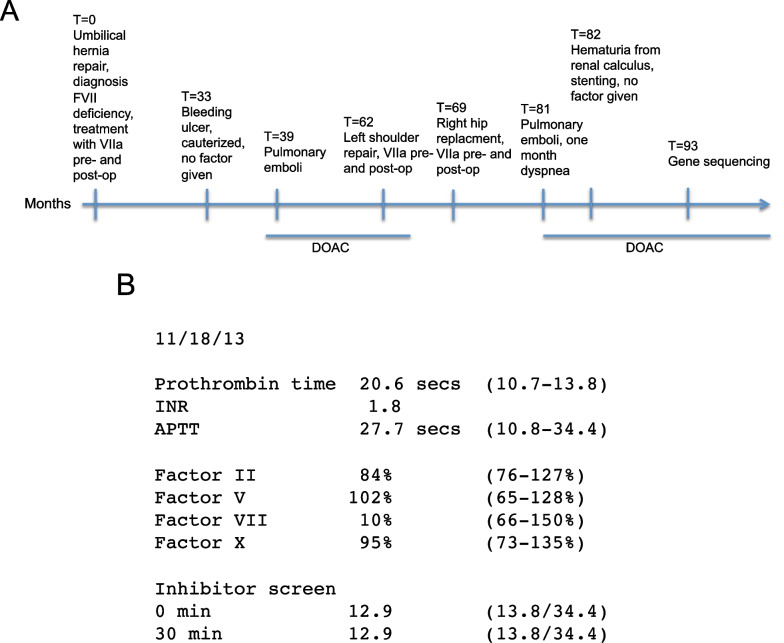
Clinical data. **A** Time line. Major events of the clinical history are arrayed on a time line calibrated in months, beginning in 2013 (*T* = 0) and ending with the present in 2022. Note in particular that bleeding episodes (ulcer *T* = 33, renal calculus *T* = 82) were provoked whereas clotting episodes (pulmonary emboli *T* = 39, pulmonary emboli *T* = 81) were unprovoked. DOAC stands for direct oral anticoagulant, a Xa inhibitor. FS has been on apixaban 5 mg twice a day continuously since *T* = 81, and he has not sustained any instances of clotting since then. **B** Special Coagulation Laboratory Data Citrated anticoagulated blood was analyzed by standard methods and PT was found to be prolonged from 10.7 to 13.8 to 20.6 s. Mixing studies revealed lack of correction with FVII deficient plasma, indicating that the patient has deficiency of FVII. No inhibitor was detected, since PT remained at 12.9 s after 1:1 mix with normal plasma and 30 min incubation at 37 degrees

In August 2016, FS developed a bleeding ulcer in the stomach. No blood products were given but endoscopy was performed, and the ulcer was cauterized. No further problems were encountered at this time. Then in early 2017, he developed dyspnea that worsened over one month. Work-up in the emergency room revealed an elevated d-dimer, and CT angiogram showed right lobar segmental and subsegmental pulmonary artery branches occluded by clot in addition to other clots on the left side of the lung without right heart strain. He was treated with low molecular weight heparin (Lovenox) 1 mg/kg every 12 h for 2 days and switched to apixaban lead-in 10 mg twice a day for 6 days followed by apixaban 5 mg twice a day. He remained on apixaban from February 2017 to February 2018, and then it was stopped (Fig. 2). The decision to stop the apixaban was based on our assessment, in retrospect incorrect, that the pulmonary emboli were provoked by inactivity and testosterone use. He had been spending 10 h per day sitting on the couch, and he was also taking testosterone cypionate 400 mg IM every 2 weeks for body building purposes. We reasoned that if the pulmonary emboli were provoked by these environmental factors then they would be unlikely to recur if the circumstances were different [10].

Mr. FS did well for a while and subsequently he had more surgeries. On 1/18/19 he had a left shoulder rotator cuff repair. Factor VIIa (NovoSeven) again was given in low doses (1 mg) pre- and post-op, and no complications ensued, either bleeding or clotting. A second surgery was performed on 7/2/19, a right hip replacement, again with factor replacement, and no complications resulted. Approximately one year later in the summer of 2020, Mr. FS developed dyspnea. This was progressively worse over one month. He was not sedentary during this time, and he was not using testosterone or other potentially procoagulant substances, and he had not received NovoSeven for over one year. Nonetheless he was diagnosed with pulmonary emboli on Computed Axial Tomography scan (CT) angiogram of 7/23/20. The report indicated numerous segmental and subsegmental non-occlusive pulmonary emboli involving both lungs from the branches distal to the right and left main pulmonary arteries involving right upper, middle, and lower lobes as well as left upper and left lower lobe. The pulmonary emboli were presumed to be unprovoked since no environmental cause could be discerned [10]. He was restarted on the DOAC, apixaban, 5 mg twice a day, because this is standard therapy for unprovoked pulmonary embolus, and since it is a factor X inhibitor, it would not be expected to exacerbate the FVII deficiency. He did well, dyspnea resolved, and evidence of clots completely dissipated on CT angiogram of 11/25/20. Subsequently he developed hematuria due to an obstructing left renal calculus. Although he was bleeding and on anticoagulation, the anticoagulation was continued throughout this episode. The obstructing stone was removed, the ureter was stented, and no factor was given. Hematuria resolved and he did well until last month. He then developed right flank pain and hematuria again, and work-up by urology showed 4 small (less than 1 cm) calculi in the right kidney. Most likely the on and off hematuria is ascribable to renal calculi rather than FVII deficiency. No factor replacement was given to treat the hematuria, and he has continued on the DOAC. Urologic follow-up consultation is being sought at the time of writing this case report (Fig. 2).

Family history is significant in that Mr. FS has European, Cherokee, and African American origins. He also has a sister who presented with a prolonged PT, was found to have FVII deficiency, and sustained an unprovoked pulmonary embolus. Like her brother she is on a DOAC and advised to remain on this indefinitely. Her FVII gene has not been sequenced. The DOAC seems to be effective in preventing clots without serious impairment of hemostasis. We are recommending that FS remain on the DOAC indefinitely. We are not managing the sister, but we would also recommend that she remain on the DOAC indefinitely since she likely has the same procoagulant mutation in FVII.

## Nature of the patient's two FVII mutant alleles

**Fig. 3:**
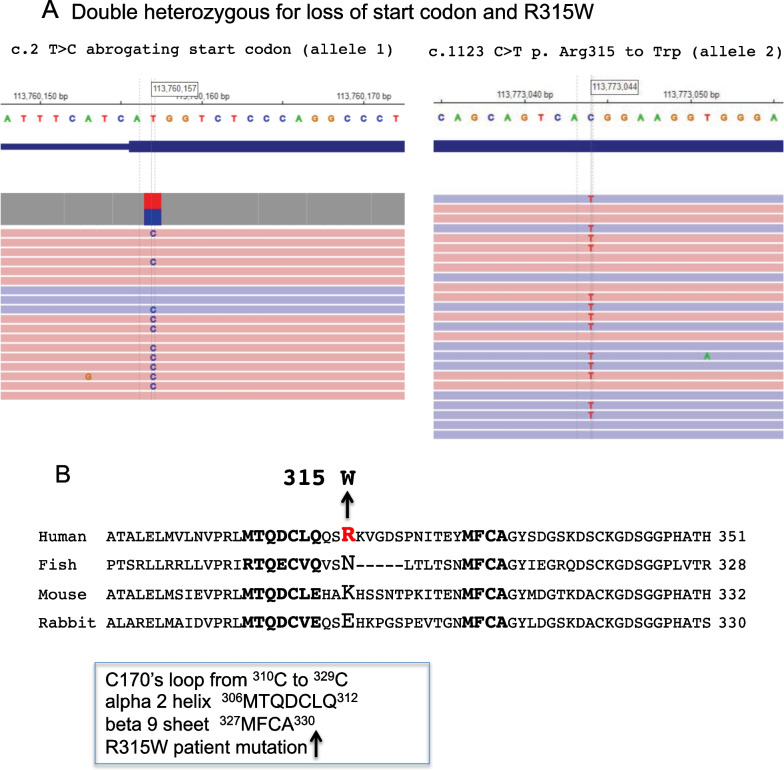
FVII Sequences** A**. Deoxyribonucleic acid sequencing of the FVII gene was performed by Fulgent Genetics using the buffy coat of a peripheral blood sample. Two mutations were detected, one c.2 T > C converted the ATG start codon to ACG, thereby abrogating the start and creating a null. A second mutation presumed to be on the other allele was detected, c.1123 C > T p. Arg 315 Trp, and this is most likely the FVII "activating" mutation.** B**. Multiple sequence alignment of the relevant region of FVII from different species. Amino acid sequences from Genbank were aligned using the multiple sequence alignment Tool Clustal W: Human Homo sapiens factor VII AAA51983.1; Fish Danio rerio factor VII precursor NP_571894.2; Mouse Mus musculus factor VII preproprotein NP_034302.2; Rabbit Coryctolagus cuniculus factor VII. Highlighted in bold capital letters is the alpha 2 helix ^306^MTQDCLQ^312^ that responds to TF binding by altering its conformation. The C170's loop extends from C310 to C329. The relevant mutation is shown by an arrow from the R315 indicated in red to the W315 above it. The beta 9 sheet ^327^MFCA^330^ is highlighted in bold capital letters

In July 2021, FS provided a blood sample for sequencing of the FVII gene, and this was performed by Fulgent Genetics. The results showed two mutations that are ascribed to two different alleles by sequencing (Fig. 3A): c.2 T > C abrogating the start codon (allele 1) and c.1123 C > T p. R315W (allele 2), introducing a missense change in amino acid 315 of the processed zymogen sequence. The allele 1 mutation changes the ATG start codon to ACG, abrogating start of translation and rendering the allele an effective null mutant. The allele 2 mutation introduces a change of amino acid 315 from R (arginine) to W (tryptophan). As can be seen from the alignment of human, fish, mouse and rabbit sequences (Fig. 3B), the mutation is predicted to occur in a critical area of the sequence of FVII, following the alpha 2 helix ^306^MTQDCLQ^312^, directly in the middle of the c170's loop, and preceding the beta 9 sheet ^327^MFCA^330^ in the linear amino acid sequence. This area of the amino acid sequence is not particularly conserved in terms of the species alignment (Fig. 3B) but is likely functionally very important. The alpha 2 helix and c170's loop (chymotrypsin numbering) function to convey the TF binding signal to the activation loop 3, thereby altering the configuration of the amino terminal serine protease region and increasing affinity for the FX substrate [11]. The effects of the allele 2 encoded protein in the context of a null allele 1 would be amplified, functioning essentially as a homozygous mutation.

## Possible mechanism of activation by mutation R315W of FVII

In the physiological setting, FVII/VIIa sits within the vascular system, anchored in the endothelium by a transmembrane domain and protruding outward with exposure to the circulation. It is mostly in the inactive zymogen form, although a small portion (about 1/100th) is cleaved at the Arg152-Ile153 peptide bond to make the heavy and light chains of VIIa. However, even this cleaved VIIa is largely inactive until exposed to TF. TF, the inducer for FVII activation, sits outside the vascular system mainly in the subendothelium segregated from FVII, and thus only if the integrity of the vascular system is disrupted does it come into contact with FVII/VIIa. In that case, though, it binds with high affinity and along a large interaction interface, inducing major conformational changes and shifts of activation loops in FVII/VIIa. The effect is to alter FVII/VIIa from a dormant state into a highly active serine protease. If the "brakes" are removed, FVII/VIIa undergoes major conformational changes that increase the affinity of the serine protease domain for its substrates. The changes include a shift of the C170's loop from an "out" position to an "in" activating position. The conformational change is transmitted to activation loop 3 and from there to the serine protease domain, which is altered in shape so that it binds substrate with more affinity and acts on its substrate with greater speed. The activated FVIIa/TF has increased activity for converting FX to Xa, the reaction proceeding with higher affinity and speed. Downstream conversions of prothrombin to thrombin, fibrinogen to fibrin, can then proceed more efficiently, leading to formation of a fibrin clot [2].

In the situation with our patient's mutant protein, FVII is sitting properly in the endothelium with no interaction with TF, which is sequestered behind the endothelial barrier. However, the conformation changes normally occurring upon FVII/VIIa upon binding TF are already partially induced by the R315W genomic mutation in TFVII/VIIa. Note the mutant form of FVII is the only form expressed since the other allele essentially expresses a null form. The mutation which occurs in the C170's loop likely produces changes in the zymogen conformation, shifting the alpha 2 helix from the "out" position to the "in" position and setting up several new interactions. The effect is to stabilize the TF-VIIa conformation over the zymogen conformation. The former lacks the "brakes" of the physiologic conformation, and is predisposed to TF interaction, thus with minimal stimulation it may become active. It is our hypothesis that the patient's FVII activity is on a hair trigger for activation, thus stimulating clotting with inappropriate minimal stimulation. In the case of FS, apparently unprovoked pulmonary emboli occurred at least twice, and further events were only prevented by continuous treatment with a DOAC.

## Conclusions

Factor VII can be considered the gatekeeper of the coagulation system [1]. Here we describe a rare inherited mutation in which the gatekeeper function is constitutively altered. Instead of the expected bleeding manifestations one might expect from low Factor VII activity, Mr. FS suffered clotting manifestations. Mr. FS did have some bleeding episodes during his clinical course, but these were always in the setting of hemostatic stress (see bleeding ulcer 8/2016 and hematuria from renal calculus 7/30/20) (Fig. 3). On the other hand, the clotting manifestations were entirely unprovoked. In the clinical setting, we consider that clots are provoked, if they occur in proximity to major surgery, immobility, serious immobilizing illness, some hormone treatments, trauma [10]. In our patient FS, none of these provocations applied. He did take testosterone for a while to assist in body building, but this may have a questionable and at most a very mildly prothrombotic effect [12]. We presume that the clots were related to his mutant FVII R315W allele, which is essentially homozygous given that the other allele has a mutation in the start codon. The possible mechanism by which FVII is activated by a R315W mutation in the zymogen has been discussed at length (see above). The efficacy of the DOAC in treating and preventing clots in this unusual situation is interesting. FS has unprovoked clotting, and so some form of anticoagulation is clearly desirable. We did not want to use warfarin or vitamin K antagonists, because these would be expected to further lower FVII activity, which is already dangerously low. The DOAC apixaban is a FXa inhibitor [13], and its site of action is perfect for the situation, because it acts just downstream of the problem, which is the unauthorized activation of FVII/VIIa. The FVII/VIIa substrate FX might be converted to FXa, but FXa effects would then be blocked, preventing downstream and inappropriate activation of the clotting system.

## Data Availability

The data for this case report are located at the Corporal Michael J. Crescenz VAMC, 3900 Woodland Avenue, Philadelphia, PA 19104.
